# A Bayesian Spatio-Temporal Analysis of Malaria in the Greater Accra Region of Ghana from 2015 to 2019

**DOI:** 10.3390/ijerph18116080

**Published:** 2021-06-04

**Authors:** Elorm Donkor, Matthew Kelly, Cecilia Eliason, Charles Amotoh, Darren J. Gray, Archie C. A. Clements, Kinley Wangdi

**Affiliations:** 1 Department of Global Health, Research School of Population Health, Australian National University, Canberra, ACT 2601, Australia; matthew.kelly@anu.edu.au (M.K.); darren.gray@anu.edu.au (D.J.G.); kinley.wangdi@anu.edu.au (K.W.); 2 Greater Accra Regional Health Directorate, Ghana Health Service, P.O. Box 184, Accra, Ghana; amotohcharles@gmail.com; 3 Department of Adult Health, School of Nursing and Midwifery, College of Health Sciences, University of Ghana, Legon, P.O. Box LG43, Accra, Ghana; celiason@ug.edu.gh; 4 Faculty of Health Sciences, Curtin University, Perth, WA 6102, Australia; archie.clements@curtin.edu.au; 5 Telethon Kids Institute, Nedlands, WA 6009, Australia

**Keywords:** Ghana, malaria, Bayesian, modelling, climatic

## Abstract

The Greater Accra Region is the smallest of the 16 administrative regions in Ghana. It is highly populated and characterized by tropical climatic conditions. Although efforts towards malaria control in Ghana have had positive impacts, malaria remains in the top five diseases reported at healthcare facilities within the Greater Accra Region. To further accelerate progress, analysis of regionally generated data is needed to inform control and management measures at this level. This study aimed to examine the climatic drivers of malaria transmission in the Greater Accra Region and identify inter-district variation in malaria burden. Monthly malaria cases for the Greater Accra Region were obtained from the Ghanaian District Health Information and Management System. Malaria cases were decomposed using seasonal-trend decomposition, based on locally weighted regression to analyze seasonality. A negative binomial regression model with a conditional autoregressive prior structure was used to quantify associations between climatic variables and malaria risk and spatial dependence. Posterior parameters were estimated using Bayesian Markov chain Monte Carlo simulation with Gibbs sampling. A total of 1,105,370 malaria cases were recorded in the region from 2015 to 2019. The overall malaria incidence for the region was approximately 47 per 1000 population. Malaria transmission was highly seasonal with an irregular inter-annual pattern. Monthly malaria case incidence was found to decrease by 2.3% (95% credible interval: 0.7–4.2%) for each 1 °C increase in monthly minimum temperature. Only five districts located in the south-central part of the region had a malaria incidence rate lower than the regional average at >95% probability level. The distribution of malaria cases was heterogeneous, seasonal, and significantly associated with climatic variables. Targeted malaria control and prevention in high-risk districts at the appropriate time points could result in a significant reduction in malaria transmission in the Greater Accra Region.

## 1. Introduction

Malaria is a preventable and treatable infectious disease caused by plasmodium parasites. The disease is commonly transmitted through the bite of an infected female *Anopheles* mosquito. Other routes include mother to child transmission, whereby infected cells from the mother are transferred to the fetus through the placenta, transfusion of infected blood to a healthy individual, donation of infected organs, and through needle stick injuries among healthcare workers and intravenous drug users [1,2,3,4]. However, these are considered minor sources of transmission. In 2019, an estimated 229 million new malaria cases and 409,000 malaria deaths occurred globally [5]. However, this burden is not distributed evenly. It is estimated that around 93% of malaria cases occur in the World Health Organization (WHO) African Region. Additionally, the group most vulnerable to malaria infection are children under five years of age, accounting for 67% of the total malaria mortality burden [5]. Malaria transmission is affected by multidimensional factors, linked to vector characteristics, climatic conditions, land cover, genetics, and human behavior [6]. These disparate factors mean that malaria transmission rates can vary significantly at local levels. Temperature and rainfall affect the life cycle and breeding of the mosquito by enabling the parasite to complete its life cycle, thus increasing the transmissibility of the disease to humans [7,8].

Similar to other endemic countries, malaria is a major cause of morbidity and is the leading cause of mortality in Ghana [9]. The country experienced half a million new cases of malaria in 2018, or 3% of all global malaria cases, and around 11,000 deaths [10]. The total number of active malaria cases is currently around 6.6 million, from a population of 29 million people. WHO guidelines for malaria control incorporate vector control measures using insecticide treated nets (ITNs) and indoor residual spraying (IRS), and prompt diagnosis and treatment. These control measures are further supported by implementation of the intermittent preventive therapy protocol (IPTp) in pregnant women [10,11]. In Ghana, the adoption of these approaches, together with increased health promotion and educational campaigns, has led to significant reductions in malaria incidence and mortality [12]. Malaria-associated mortality had been reduced by 14.8% in 2016 compared to 2010 [13]. Case fatality rates in children under five years experienced a 4% reduction within the same timeframe. In addition, the facility-based malaria fatality rate for children under five years reduced from 14% in 2000 to 0.5% in 2016 [9]. Despite these achievements, malaria remains a major health burden, and the reductions in incidence and mortality have not been sustained. In fact, there was an 8% increase in malaria cases in Ghana in 2018 compared to 2017 [10].

**Figure 1:**
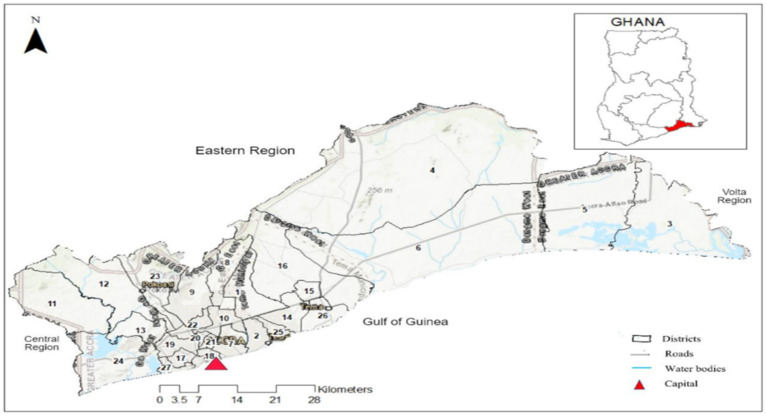
Map of Greater Accra Region with 29 districts and neighboring regions, roads, water bodies, and regional capital. Source: Authors’ own contribution.

As in other settings, the effects of climatic variables and location on malaria transmission are well established in Ghana [14]. In addition, social and economic factors affect the incidence of malaria in the country [14,15]. In terms of location, malaria prevalence varies significantly by region and tends to be higher in rural localities than in urban areas [9]. For instance, the Greater Accra Region had the lowest malaria prevalence (5%) among children under five years in the country in 2016, in contrast to the northern region (25%) and eastern and central regions (both with 31%) [15]. This heterogeneity is influenced by several factors such as the immune profile of populations, stability of mosquito breeding places (related to climate factors), varying density of populations, and accessibility to public health interventions and healthcare [16,17]. As a result, policies targeting malaria prevention and control need to be designed specifically for regional and local levels, utilizing local specific data rather than national-level aggregated data, which may not reflect disease distribution at the regional and local levels. The aim of this study was to assess the inter-district variation of malaria burden and the climatic drivers of malaria in the Greater Accra Region (Figure 1), where malaria is among the top five infections reported at healthcare facilities [18], using spatio-temporal statistical and analytical techniques.

## 2. Materials and Methods

### 2.1. Description of the Study Area

The study was conducted in the Greater Accra Region, which includes the country’s capital city, Accra (Figure 1). It is the smallest of the 16 administrative regions in Ghana. The area is about 3245 square kilometers. It is the second-most populated region in the country with a population of 5,055,765 in 2019 [18]. It is divided into 29 local government areas (LGAs), namely: 1—Adenta Municipal, 2—Ledzokuku Municipa, 3—Ada East, 4—Shai Osudoku, 5—Ada West, 6—Ningo/Prampram, 7—La Dade-Kotopon, 8—La-Nkwantanang-Madina, 9—Ga East, 10—Ayawaso West, 11—Ga South Municipal, 12—Ga West Municipal, 13—Ga Central Municipal, 14—Tema West Municipal, 15—Ashaiman Municipal, 16—Kpone Katamanso, 17—Ablekuma Central Municipal, 18—Korle Klottey Municipal, 19—Ablekuma North Municipal, 20—Ayawaso North Municipal, 21—Ayawaso East Municipal, 22—Okaikwei North Municipal, 23—Ga North Municipal, 24—Weija Gbawe Municipal, 25—Krowor Municipal, 26—Tema Metropolitan, 27—Ablekuma West Municipal, 28—Ayawaso Central Municipal, and 29—Accra Metropolis (Figure 1). Most of the LGAs are urban, and urban LGAs house more than 80% of the region’s residents [19]. The LGAs are classified as districts, municipalities, and metropolitan areas, with metropolitan areas being the most urban. However, for the purpose of this study, they are all referred to as districts. The blend of remote and urban communities within the region makes it a suitable site for this study. The climate of the area is categorized as tropical savannah with high humidity and high temperatures.

### 2.2. Data Sources

The study used secondary aggregated clinical data for all age groups, including both inpatients and outpatients, with confirmed malaria infections (rapid diagnostic test and microscopy) from 2015 to 2019 in the Greater Accra Region. All malaria cases were identified through passive case detection at health facilities and collated in the DHIMS at the district level. Yearly district population was obtained from the Health Information Department of the Greater Accra Regional Health Directorate. 

Long-term average annual and seasonal temperature and rainfall variables were determined using data obtained from the WorldClim project at a spatial resolution of 1 km [20]. The variables obtained from WorldClim had been created by spatial interpolation of climate data gathered from global weather station sources between 2010 and 2018 by utilizing a thin plate smoothing spline algorithm.

Polygon shapefiles of administrative boundaries at the district level of the Greater Accra Region were obtained from the DIVA-GIS website [21]. The spatial datasets, including population densities per square kilometer (PD) and standard morbidity ratios (SMR) of malaria cases, were imported into ArcGIS version 10.7.1 software [22] and projected to the Universal Transverse Mercator (UTM) coordinate system (zone 48 N).

- A seasonal-trend decomposition, based on locally weighted regression (STL) was used to decompose the time series of malaria incidence to reveal the seasonal relationship, inter-annual pattern, and the residual variability. The STL model was structured as follows:
  (1) $$Y_{t}=S_{t}+T_{t}+R_{t}$$
  where *Y_t_*, *S_t_*, *T_t_*, and *R_t_* represent the local malaria cases with logarithmic transformation, additive seasonal component, trend, and remainder component, respectively, while *t* signifies time in months [23,24,25].
- Standardized morbidity ratios (SMRs) per district were analyzed using the following formula:
  (2) $$Y_{i}=\frac{O_{i}}{E_{i}}\times 100$$
  where *Y* denotes the total SMR in district *i*, and *O* and *E* are, respectively, the total number of the observed and expected malaria cases in district *i* across the study period. The expected number (*E*) was calculated by multiplying the regional malaria incidence by the average population for each district over the study period.

*Annual Parasitic Incidence* (*API*) per district were calculated using the formula:

(3) $$Annual\;Parasitic\;Incidence\;\left(API\right)=\frac{Total\;No.\;of\;Malaria\;Cases\;in\;a\;Year\;}{Total\;Population}\times 1000$$

### 2.3. Independent Climatic Variable Selection

A preliminary negative binomial (NB) regression was used to select the significant climatic covariates. Maximum and minimum temperature and rainfall with zero-, one-, two-, three-, four-, five-, and six-month lag times were entered into univariate NB models. The most significant (*p* < 0.05) climatic variables with the lowest Akaike’s information criterion (AIC) were selected for inclusion in the model (Table S1). The co-linearity of the selected variables was tested using variance inflation factors (VIF). Minimum temperature without lag, rainfall lagged at one month, and maximum temperature lagged at six months were selected (Table S2). Preliminary statistical analyses were all performed using STATA software, version 16.0 [26].

### 2.4. Spatio-Temporal Model

NB regression was selected over Poisson regression because of the overdispersion and lower Akaike’s information criterion and Bayesian information criterion (Table S3). NB models were created via a Poisson–Gamma distribution structure using the Bayesian statistical software WinBUGS, version 1.4 [27] (Table S4). Three models were created, incorporating spatially unstructured (Model I), spatially structured (Model II), and both structured and unstructured random effects (Model III). Each model included the climatic variables as fixed effects. The best-fit parsimonious model was selected with the lowest deviance information criterion (DIC). Model III, which includes all components of the other models, was structured as follows:

*Y_ij_* ~ Poisson (*μ_ij_*) (4)

log (*μ_ij_*) = log (*E_ij_*) + *θ_ij_* (5)

*θ_ij_* = *α* + *β*_1_ × *trend_j_* + *β*_2_ × *rainfall_ij_* + *β*_3_ × *max temp_ij_* + *β*_4_ × *min temp_ij_* + *u_i_* + *s_i_* + *w_ij_* (6)

and the dispersion parameter were defined as:

(7) $$\tau_{i}\sim Г\left(\alpha ,\alpha \right)$$

(8) α=exp(logα)

(9) logα~Normal(0,0.01)

where *Y* is the observed count of malaria for the *i*th district (*i* = 1…60) in the *j*th month (January 2015 to December 2019), *E* is the expected number of malaria cases included as an offset to control for population size, and *θ* is the mean log relative risk (RR); *α* is the intercept and *β*_1_, *β*_2_, *β*_3_, and *β*_4_ are the coefficients for monthly malaria trend, rainfall lagged at one month, maximum temperature lagged at six months, and minimum temperature without lag, respectively. The unstructured and spatially structured random effects are represented by *u_i_* and *s_i_*, each with a mean of zero and with variances of σ*_u_*^2^ and σ*_s_*^2^ and w*_ij_* is the spatio-temporal random effect (with a mean of zero and variance of σ_w_^2^).

The spatially structured random effect was calculated using a conditional autoregressive (CAR) prior structure. Spatial relationships between the districts were computed using queen contiguity, where an adjacency weight of 1 was allocated if two districts shared a common border or vertex and 0 if they did not. The intercept was delineated with a flat prior distribution, while the coefficients were defined by a normal prior distribution. Non-informative gamma distributions, characterized by shape and scale parameters equivalent to 0.01, were used to specify priors for the precision (1/σ*_u_*^2^ and 1/σ*_s_*^2^) of the unstructured and spatially structured random effects. Additionally, models were established without the structured (Model I) and unstructured (Model II) random effects to determine if including them improved model fit.

The burn-in, comprising the initial 10,000 iterations, were discarded. The simulation chains were then run for blocks of 20,000 iterations to assess for convergence. Convergence was determined through visual inspection of posterior density and history plots for each model and was achieved at 100,000 iterations. Markov Chain Monte Carlo simulation with Gibbs sampling was used to estimate model parameters [28]. Values of the posterior distributions were then stored and summarized for analysis (posterior mean and 95% credible intervals (CrI)).

An α-level of 0.05 was used to indicate statistical significance (as shown by 95% CrI for coefficients (*β*) that excluded 0). ArcMap 10.5 software (ESRI, Redlands, CA, USA) was used to produce maps [22].

## 3. Results

### 3.1. Descriptive Analysis

**Figure 2:**
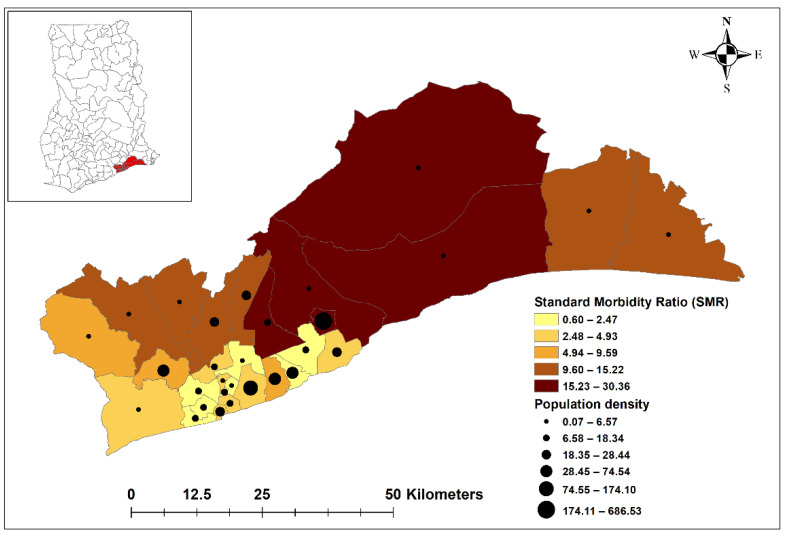
Standard morbidity ratios (SMRs) and population densities per square km by district in the Greater Accra Region, 2015–2019. Source: Authors’ own contribution.

A total of 1,105,370 malaria cases were recorded in the region during the study period (2015–2019). The overall malaria incidence was approximately 47 per 1000 population at risk. All districts recorded malaria cases during the study period. Malaria transmission was spatially heterogeneous across the study area, with Ashaiman district recording the highest number of cases (187,322, 16.9%) and Ayawaso West district the lowest (1739, 0.2%) (Figure 2). Ashaiman district had the highest API of 168.8, while Ablekuma Central district recorded the lowest API of 3.4 (Table 1). Generally, higher rates of malaria infection were observed in the north-eastern districts with low population densities, including Shai Osudoku, Ningo Prampram, Kpone Katamanso, Ashaiman, and Adenta, whereas districts with higher population densities, located in the south-western part of the study area, were associated with lower malaria incidence (Figure 2). The highest rainfalls were experienced in June, while maximum temperatures were experienced in February. January was the month with the lowest minimum temperatures during the study period, except for 2016 and 2017 when August had the lowest minimum temperatures (Table 2).

### 3.2. Time Series Decompositions

**Figure 3:**
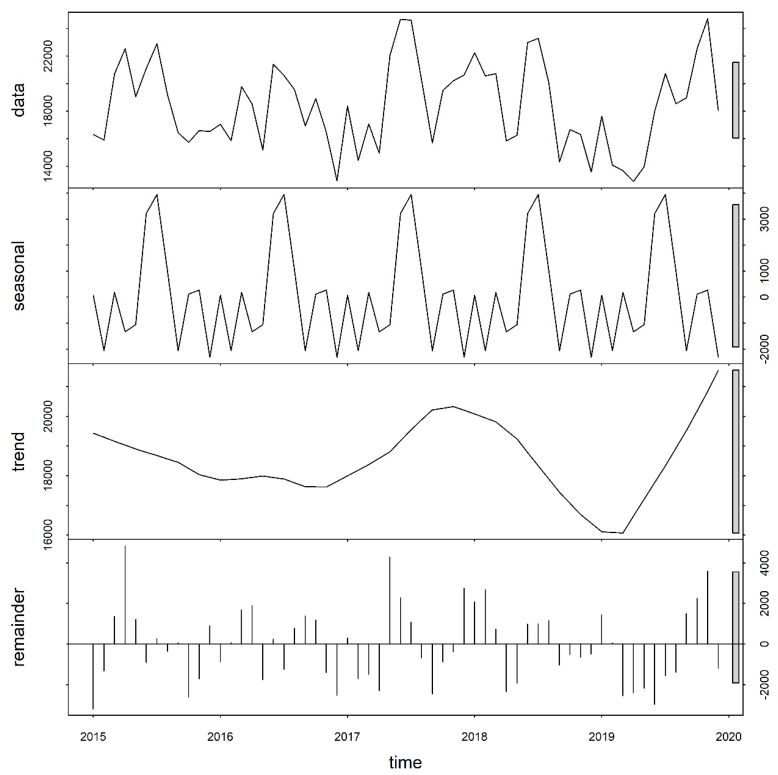
Temporal decomposition of numbers of malaria cases for the Greater Accra Region, 2015–2019. Source: Authors’ own contributions.

Time series decomposition analysis revealed seasonal patterns of malaria cases during the study period. Four peaks were observed, with the largest peak occurring in June of 2016 and 2017, and in July of 2015 2018, and 2019. The three smaller peaks occurred in March, September, and October throughout the study period. The inter-annual pattern indicated a general increasing trend of cases, but with sharp fluctuations (Figure 3).

### 3.3. Negative Binomial Regression Analysis

**Figure 4:**
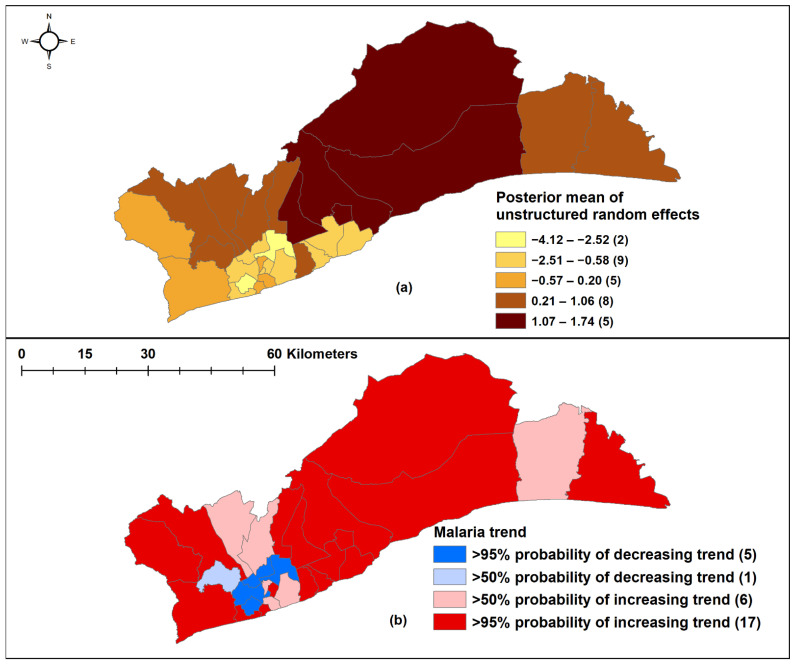
Spatial negative binomial regression analysis. (**a**) Unstructured random effects of malaria in Model I; (**b**) Trend analysis during the study period, 2015–2019. Source: Authors’ own contributions.

Model I, with unstructured random effects, was the best-fit, most parsimonious model (as indicated by the lowest DIC). Monthly malaria cases increased by 22.9% (95% CrI: 19.6–25.6%) per month during the study period. Malaria incidence decreased by 2.3% (95% CrI: 0.7–4.2%) for each 1 °C increase in monthly mean minimum temperature without lag. Monthly rainfall and maximum temperature were not statistically significant in predicting malaria cases (Table 3 and Figure 4a).

In 17 districts, there was a >95% probability of a higher than national average increasing trend, while five districts had a >95% probability of monthly malaria trend lower than the regional average, and these were in the south-central part of the region (Figure 4b).

## 4. Discussion

This study assessed the inter-district variation of malaria burden and the climatic drivers of malaria in the Greater Accra Region of Ghana. Malaria incidence varied across the region and increased over the study period with a strong seasonal pattern. The significant covariate of malaria transmission was monthly minimum temperature without lag.

The incidence of malaria was found to vary significantly between districts, with only five out of the 29 districts, located in the southcentral area, having a lower trend than the regional average trend. Additionally, less urbanized districts were found to be associated with more cases and higher disease risk compared to less urbanized districts. Previous studies have reported that lower incidence of malaria in more urban areas was related to greater social and economic development, including access to better housing and drainage systems as well as more accessible healthcare, resulting in decreased human vector contact and more active case management [29,30,31]. Populations in less urbanized areas are also more likely to be engaged in agricultural and other occupational activities, leading to more exposure to mosquitos, which breed in these areas [16,32]. This study also showed a strong relationship between population density and malaria incidence, as seen in Figure 2. Districts with lower population densities tended to be less urbanized and correlated with a higher malaria incidence. This could be due to a lower ratio of humans to vectors in these districts, resulting in an increased biting rate [14,33,34]. 

There was a clear indication of seasonality and an irregular inter-annual pattern of malaria transmission in the Greater Accra Region; higher numbers of cases were recorded in the rainy seasons and in 2017 and 2019, respectively. A previous study by Donovan et al. [35], which utilized clinical and self-reported malaria data, also found that malaria transmission increased during the rainy season in Accra. However, rainfall lagged at one month was not found to be statistically significant in predicting malaria cases in this study, contradicting findings from previous studies [35,36]. For example, some studies found poor associations between malaria and rainfall [37,38], while others reported a strong negative association between malaria transmission and rainfall [39,40]. At the population level, lag effects are linked to factors affecting mosquito development and parasite incubation [41,42]. As such, features in urban areas, including open gutters and poor disposal of refuse, may provide breeding conditions conducive to mosquito survival and an increase in malaria risk. Additionally, wet seasons are usually associated with flooding and pooling of water. This, together with the few water bodies found in the environment, serve as breeding grounds for the malaria vector. These may have contributed significantly to the rise in malaria cases. However, the decreasing number of cases in 2016 and 2018 could be due to the protection offered from the mass distribution of LLIN in those years, but LLIN was not included in our model. 

The model results from this study showed that increasing monthly maximum temperature negatively correlated with malaria incidence. This association was not statistically significant (RR: 0.996, 95% CrI: 0.969–1.007). However, the effects of increasing temperature on vector populations, the incubation period of malaria parasites, and malaria transmission are well-known [43,44]. Increasing temperature promotes malaria transmission rates and extends malaria geographically [45]. Optimal temperature values from 23 °C to 31 °C usually favor malaria parasite development and vector survival, resulting in an increased malaria incidence [46]. Temperature values above the optimal range have negative effects on the malaria vector and parasite, leading to reduced malaria incidence. Additionally, there is general recognition of the relationship between lagged climatic variables and malaria incidence over time. For instance, in a systematic review by Reiner Jr. et al. [47] on the seasonality of *Plasmodium falciparum* transmission, lagged temperature varying from zero month to nine months were observed to drive global malaria incidence significantly. Individual or a combined influence of factors, including habitat formation, vector development, infectivity, and emergence of signs and symptoms in humans during the lag’s periods, affects malaria transmission dynamics [48,49,50].

It is uncertain why an increase in minimum temperature was associated with reduced malaria incidence during the study period. Minimum temperature was also found to affect the transmission of malaria in previous studies [51,52]. One would expect an increase in minimum temperature to decrease the extrinsic incubation period (EIP) for *P. falciparum* [53,54,55,56], thereby, increasing malaria transmission. However, the complex interactions among vector, parasitic, behavioral, socioeconomic, and healthcare factors not included in the model may have contributed to the results observed.

Findings from this study need to be interpreted with the consideration of some limitations. Firstly, district by district data were not available to measure and compare the availability, accessibility, and usage of malaria control and prevention strategies (LLIN, IPTp, IRS), which could have potentially influenced malaria transmission in the Greater Accra region. Hence, these factors were not included in the model. Secondly, the study used passive surveillance data, for which the quality and reliability could not be readily ascertained. Thirdly, unmeasured risk modifiers, including living standards and socio-economic development, treatment seeking behaviors, and population mobility, were not accounted for in this study. Lastly, consideration was not given to individuals who sought care in healthcare facilities outside their districts of residence because of personal preferences or proximity. The limitations discussed above were suggested to have influenced the pattern of diseases, including malaria transmission in previous studies [25,57,58,59]. However, our results agree with prior studies on the relation between climate and malaria [47]. Malaria transmission within the region may have been mediated by multiple factors apart from the climatic covariates assessed, including vector characteristics and environmental and human population dynamics [48,50]. Hence, future studies should use methods that will explore both linear and non-linear relationships between climatic conditions and malaria incidence within the study region. 

## 5. Conclusions

Findings from this study showed a negative correlation between malaria and minimum temperature in the Greater Accra Region of Ghana from 2015 to 2019. Additionally, malaria transmission was heterogeneous across the districts and showed strong seasonal and inter-annual variations. The results reported in this study expand on knowledge regarding the influence of climatic factors on malaria transmission previously found in other parts of Ghana, sub–Saharan Africa, and other malaria endemic countries [14,33,37,47]. Our study also highlighted the variations in malaria incidence at a small geographical scale and shows the relevance of using modelling and geographical information systems methods to explore the spatial and temporal patterns of malaria transmission. It is also relevant to mention the interactions between malaria and COVID-19 in the current era. In resource constrained low- and middle-income countries, including Ghana, the COVID-19 pandemic has the potential to undermine targeted malaria control efforts based on our findings because of overlapping symptoms, and because of diversion of limited public health resources from malaria control to COVID-19 response [60,61,62]. Although the transmission pattern identified in our study could be influenced by other variables not considered, our findings showed that malaria is still a disease of great importance in Ghana. Hence, there is a need to continue with malaria control and prevention interventions alongside COVID-19 measures in general to ensure that the malaria situation does not worsen post COVID-19. Most importantly, malaria control measures should be intensified in the high-risk districts by increasing LLINs, IRS, and IPTp, especially during periods during which climatic variables assessed were identified to be associated with high malaria transmissions in the region. 

## Figures and Tables

**Table 1:** Total malaria cases and API stratified by 29 districts (2015–2019).

No.	Districts	Total Malaria Cases	Percentage	API *
1	Adenta Municipal	69,758	6.3	156.1
2	Ledzokuku Municipal	39,394	3.6	49.7
3	Ada East	32,175	3.0	78.0
4	Shai Osudoku	48,989	4.4	164.2
5	Ada West	23,941	2.2	70.1
6	Ningo/Prampram	66,714	6.0	162.7
7	La Dade-Kotopon	17,437	1.6	16.4
8	La-Nkwantanang-Madina	51,889	4.7	80.2
9	Ga East	68,924	6.2	80.1
10	Ayawaso West	1739	0.2	4.0
11	Ga South Municipal	57,602	5.2	36.3
12	Ga West Municipal	54,642	5.0	77.0
13	Ga Central Municipal	36,113	3.3	53.3
14	Tema West Municipal	6369	0.6	9.6
15	Ashaiman Municipal	187,322	16.9	168.8
16	Kpone Katamanso	93,987	8.5	148.3
17	Ablekuma Central Municipal	3355	0.3	3.4
18	Korle Klottey Municipal	20,227	1.8	26.5
19	Ablekuma North Municipala	11,356	1.0	12.6
20	Ayawaso North Municipal	10,142	0.9	22.4
21	Ayawaso East Municipal	5981	0.5	9.9
22	Okaikwei North Municipal	21,544	1.9	18.0
23	Ga North Municipal	49,129	4.4	84.6
24	Weija Gbawe Municipal	25,038	2.3	27.4
25	Krowor Municipal	6241	0.6	11.8
26	Tema Metropolitan	16,993	1.5	16.5
27	Ablekuma West Municipal	13,122	1.2	13.7
28	Ayawaso Central Municipal	7523	0.7	6.5
29	Accra Metropolis	57,724	5.2	22.3
	Total	1,105,370	100	1630.1

* API-annual parasite incidence per 1000 population.

**Table 2:** Monthly average rainfall (mm), average maximum temperature (°C), and average minimum temperature (°C) in the Greater Accra Region from 2015 to 2019.

Month	Average Rainfall					Average Max. Temperature					Average Min. Temperature				
	2015	2016	2017	2018	2019	2015	2016	2017	2018	2019	2015	2016	2017	2018	2019
Jan.	6.5	9.2	21.7	4.7	4.7	32.4	33.5	33.5	33.0	33.0	23.0	23.8	23.5	23.0	23.0
Feb.	66.0	15.8	19.7	36.8	36.8	33.7	34.8	34.2	34.0	34.0	24.7	25.1	24.5	24.7	24.7
Mar.	121.6	96.9	68.1	74.7	74.7	33.8	34.0	34.2	33.1	33.1	24.5	24.9	24.8	24.2	24.2
Apr.	83.5	98.9	88.7	84.6	84.6	33.7	33.5	33.6	33.3	33.3	24.7	25.3	25.0	24.6	24.6
May	120.4	175.2	166.6	137	136.9	32.8	32.6	32.1	32.4	32.4	24.6	24.6	24.5	24.4	24.4
Jun.	211.1	195.4	314.9	178	178.3	30.6	30.2	30.2	30.4	30.4	24.1	23.9	23.9	24.0	24.0
July	45.9	52.8	72.5	67.8	67.8	29.3	29.2	29.4	29.3	29.3	23.2	23.3	23.3	23.3	23.3
Aug.	34.0	31.8	41.2	45.0	45.1	29.0	29.0	28.6	29.0	29.0	23.0	23.0	22.6	23.0	23.0
Sept.	64.7	102.5	82.5	115.1	115.1	30.2	29.9	29.9	30.1	30.1	23.3	23.5	23.2	23.3	23.3
Oct.	120.0	111.7	65.2	145.5	145.5	31.5	31.8	32.0	31.5	31.5	23.7	23.9	23.9	23.8	23.8
Nov.	77.1	99.0	119.5	50.6	50.6	33.0	33.1	33.0	32.8	32.8	24.1	24.5	24.1	24.2	24.2
Dec.	11.6	44.0	28.7	26.1	26.1	33.0	34.0	33.2	32.9	32.9	23.7	24.7	24.1	23.5	23.5

**Table 3:** Regression coefficients, relative risk, and 95% credible interval from Bayesian spatial and non-spatial models of malaria in the Greater Accra Region, Ghana, January 2015–December 2019.

Model/Variable	Coeff, Posterior Mean (95% CrI)	RR, Posterior Mean (95% CrI)
Model I (Unstructured) **		
Mean monthly trend	0.207 (0.179, 0.228)	1.229 (1.196, 1.261)
Monthly rainfall (10 mm) *	1.58 × 10^−5^ (−9.39 × 10^−5^, 4.65 × 10^−4^)	1.000 (1.000, 1.000)
Monthly maximum Temp (°C) ^⁑^	−3.55 × 10^−3^ (−3.12 × 10^−2^, 6.78 × 10^−3^)	0.996 (0.969, 1.007)
Monthly minimum Temp (°C)	−2.30 × 10^−3^ (−4.24 × 10^−2^, −7.34 × 10^−3^)	0.977 (0.958, 0.993) ^a^
Heterogeneity		
Structured (trend)	0.503 (0.267, 0.816)	
Unstructured	0.502 (0.270, 0.809)	
DIC	16,563.2	
Model II (Structured)		
Mean monthly trend	0.261 (0.254, 0.268)	1.231 (1.202, 1.260)
Monthly rainfall (10 mm) *	−1.33 × 10^−5^ (−1.04 × 10^−4^, 7.467 × 10^−5^)	1.000 (1.000, 1.000)
Monthly maximum Temp (°C) ^⁑^	−1.59 × 10^−3^ (−1.17 × 10^−2^, 8.6 × 10^−3^)	0.998 (0.988, 1.009)
Monthly minimum Temp (°C)	−2.17 × 10^−2^ (−3.76 × 10^−2^, −5.22 × 10^−3^)	0.978 (0.963, 0.995)
Heterogeneity		
Structured (trend)	0.508 (0.272, 0.828)	
Structured (spatial)	0.116 (0.064, 0.184)	
DIC	16,589.8	
Model III (Mixed)		
Mean monthly trend	0.207 (0.182, 0.232)	1.230 (1.200, 1.261)
Monthly rainfall (10 mm) *	−5.35 × 10^−6^ (−1.06 × 10^−4^, 8.20 × 10^−5^)	1.000 (0.9999, 1.0001)
Monthly maximum Temp (°C) ^⁑^	−1.98 × 10^−3^ (−1.30 × 10^−2^, 9.56 × 10^−3^)	0.998 (0.9871, 1.0096)
Monthly minimum Temp (°C)	−2.18 × 10^−2^ (−3.92 × 10^−2^, −5.53 × 10^−3^)	0.978 (0.9616, 0.9945)
Heterogeneity		
Structured (trend)	0.504 (0.269, 0.821)	
Unstructured	0.951 (0.423, 2.236)	
Structured (spatial)	1.590 (0.153, 5.376)	
DIC	16,579.0	

^a^ significant; ** best-fit model; * lagged one month, ^⁑^ lagged six months. Abbreviations: coeff—coefficients; CrI—credible interval; RR—relative risk; DIC—deviation information criterion.

## Data Availability

The datasets generated and/or analyzed during the current study are available from the corresponding author on reasonable request.

## Supplementary Materials

The following are available online at https://www.mdpi.com/article/10.3390/ijerph18116080/s1. Supplementary Table S1: Univariate preliminary analysis of variables using negative binomial regression. Supplementary Table S2: Variance inflation factor (VIF) for collinearity of the selected variables. Supplementary Table S3: Model comparison using Akaike’s information criterion and Bayesian information criterion. Supplementary Table S4: WinBugs codes for negative binomial mixed model.

## Abbreviations

AIC	Akaike’s information criterion
API	Annual parasitic incidence
BIC	Bayesian information criterion
CAR	Conditional autoregressive
COVID-19	Coronavirus disease 2019
CrI	Credible interval
DHIMS	District Health Information and Management System
DIC	Deviance information criterion
EIP	extrinsic incubation period
GAR	Greater Accra Region
LGAs	Local Government Areas
IPTp	Intermittent Preventive Treatment in pregnancy
IRS	indoor residual spraying
LLIN	long lasting insecticide net
MCMC	Markov chain Monte Carlo
NB	Negative Binomial
RR	Relative risk
SMR	Standardized morbidity ratios
STL	Seasonal-trend decomposition, based on locally
VIF	Variance inflation factors
WHO	World Health Organization
