# Supplementary material for: A Bayesian Spatio-Temporal Analysis of Malaria in the Greater Accra Region of Ghana from 2015 to 2019
Source: Int J Environ Res Public Health. 2021 Jun 4;18(11):6080. doi: 10.3390/ijerph18116080 (PMC8200193; doi:10.3390/ijerph18116080)
Supplement: Supplementary file 1 [file ijerph-18-06080-s001.zip › ijerph-1205190-supplementary.pdf]

Supplementary material

Supplementary Table 1 Univariate preliminary analysis of variables using negative binomial regression.

Variables	IRR	95% CI	p value	AIC	BIC
**Without lag**					
Rainfall	1.00	1.0002, 1.002	0.01	25868.57	25884.95
Max Temp	0.967	0.939, 0.996	0.026	25870.33	25886.72
Min Temp	0.837	0.7783, 0.9008	<0.000	25852.76	25869.15
**One-month lag**					
Rainfall	1.002	1.0011 - 1.0029	<0.000	25838.05	25854.43
Max Temp	0.987	0.9581, 1.0168	0.389	25856.85	25873.24
Min Temp	0.91	0.8455, 0.9801	0.013	25851.37	25867.75
**Two months lag**					
Rainfall	1.001	1.0005 - 1.0023	0.003	25831.47	25847.86
Max Temp	1.005	0.976, 1.035	0.741	25840.67	25857.05
Min Temp	0.94	0.871, 1.001	0.065	25837.36	25853.74
**Three months lag**					
Rainfall	1.001	0.9996, 1.0014	0.288	25820.33	25836.71
Max Temp	1.018	0.989, 1.048	0.23	25820.04	25836.42
Min Temp	0.94	0.876, 1.01	0.09	25818.6	25834.98
**Four months lag**					
Rainfall	1.001	0.9995, 1.0014	0.333	25802.62	25818.99
Max Temp	1.027	0.998, 1.057	0.072	25800.36	25816.74
Min Temp	0.95	0.887, 1.024	0.189	25801.84	25818.22
**Five months lag**					
Rainfall	1.000	0.9993, 0.0010	0.748	25786.86	25803.24
Max Temp	1.032	1.0026, 1.0628	0.033	25782.43	25798.81
Min Temp	0.92	0.857, 0.989	0.024	25781.89	25798.27
**Six months lag**					
Rainfall	1.000	0.9989, 1.0006	0.585	25769.00	25785.37
Max Temp	1.033	1.0027, 1.0636	0.032	25764.76	25781.13
Min Temp	0.9	0.838, 0.968	0.004	25761.16	25777.53

Supplementary Table 2 Variance inflation factor (VIF) for collinearity of the selected variables

Variable	VIF
Min Temperature without lag	1.09
Rainfall 1-month lag	1.30
Max Temperature 6 months lag	1.39
**Mean VIF**	**1.26**

Supplementary Table 3 Model comparison using Akaike's information criterion and Bayesian information criterion

**Models**	**Observations**	**AIC**	**BIC**
**Poisson**	1,734	1056609	1056631
**Negative Binomial**	1,734	25734.14	25761.43

Supplementary Table 4 WinBugs codes for negative binomial mixed model

```
#Negative binomial mixed model

model {

        for (i in 1:29) {                    # number of states
        for (j in 1:60){                     # time interval

        Case[i,j]  ~ dpois(mustar[i,j])  #Negative binomial distribution

        mustar[i,j]<-rho[i,j]*mu[i,j]          #product log link


        rho[i,j] ~ dgamma(alpha,alpha) #overdispersion

        log(mu[i,j]) <- log(Exp[i,j]) + zeta[i,j]

        zeta[i,j] <- intercept + v[Dist[i]] + u[Dist[i]] + clim[Dist[i],j] + time[Dist[i],j] +
beta[1]*t[j]
                }
                }


                for(i in 1:29){
                u[i]~dnorm(0,tau.u)

                for (j in 1:60){
                        clim[i,j]<-beta[2]*Rainfall[i,j] + beta[3]*Tmax[i,j] +
beta[4]*Tmin[i,j]

                        time[i,j]<-delta[i]*t[j]
                }
                }
# Priors

            v[1:29] ~ car.normal(adj[], weights[], num[], tau.v)
```

```
for(j in 1:128) { weights[j] <- 1}

delta[1:29] ~ car.normal(adj[], weights[], num[], tau.delta)

for (i in 1:4){
            beta[i] ~ dnorm(0.0, 0.00001)
            }

intercept~dflat()
tau.u~dgamma(0.5, 0.001)
tau.v~dgamma(0.5, 0.001)
tau.delta~dgamma(0.001, 0.001)

alpha<-exp(logalpha)
logalpha~dnorm(0,0.01)


            }
```

# Initials

```
list(
intercept=0,
beta=c(0.5, 0.5, 0.5, 0.5),          # 4 beta so 4 values imputed
tau.u=0.5,
tau.v=0.5,
tau.delta = 0.5,
)
```

##
